# 5,7-Dihydr­oxy-3,6,8-trimethoxy­flavone

**DOI:** 10.1107/S1600536809050715

**Published:** 2009-11-28

**Authors:** Hui-Ping Xiong, Zhi-Jun Wu, Fa-Tang Chen, Wan-Sheng Chen

**Affiliations:** a Department of Mathematics and Physics, Shanghai University of Electric Power, Shanghai 200090, People’s Republic of China; b Department of Pharmacy, Changzheng Hospital, Second Military Medical University, Shanghai 200003, People’s Republic of China

## Abstract

The title compound (systematic name: 5,7-dihydr­oxy-3,6,8-trimeth­oxy-4*H*-chromen-4-one), C_18_H_16_O_7_, is a flavone that was isolated from *Ainsliaea henryi*. There are two mol­ecules in the asymmetric unit, one of which has a disordered meth­oxy group [occupancy ratio 0.681 (9):0.319 (9)]. Both mol­ecules have an intra­molecular O—H⋯O hydrogen bond. In the crystal, mol­ecules are linked into O—H⋯O hydrogen-bonded chains parallel to [110].

## Related literature

For similar compounds and background information, see: Chinese Materia Medica (2007 ▶); Ali *et al.* (1979 ▶); Cubukcu & Bingol (1984 ▶); Guerreiro *et al.* (1982 ▶); Horie *et al.* (1995 ▶); Jakupovic *et al.* (1989 ▶); Lavault & Richomme (2004 ▶); Mericli *et al.* (1986 ▶); Torrenegra *et al.* (1980 ▶); Urzua *et al.* (1995 ▶); Wollenweber *et al.* (1993 ▶, 2008 ▶). For the anti­fungal activity of the title compound, see: Tomas-Lorente *et al.* (1989 ▶).
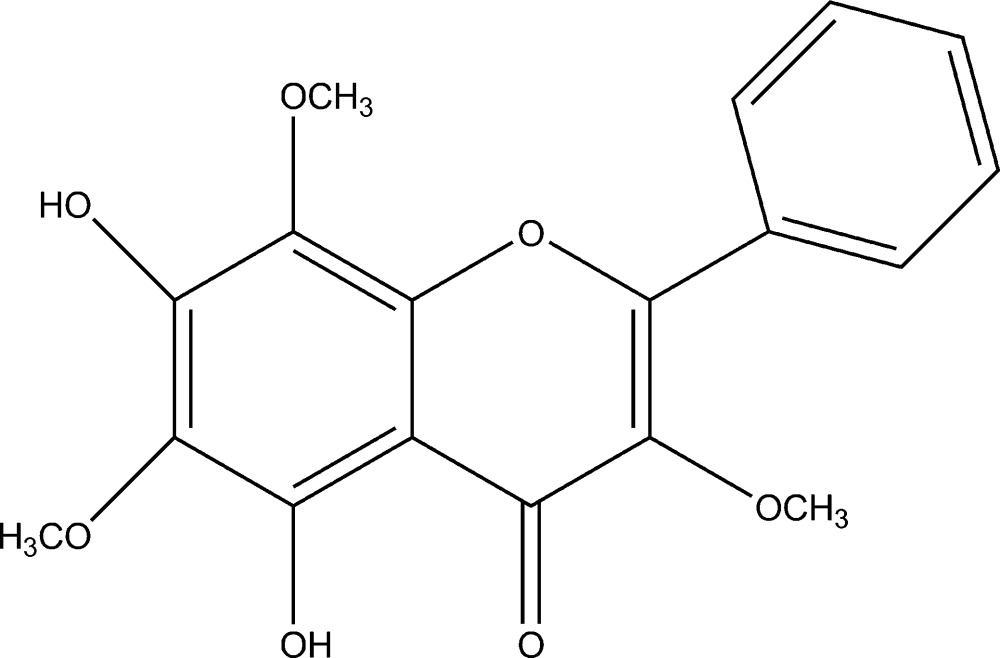


## Experimental

### 

#### Crystal data

C_18_H_16_O_7_
*M* *_r_* = 344.31
Triclinic, 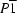

*a* = 10.147 (4) Å
*b* = 11.493 (4) Å
*c* = 14.134 (5) Å
α = 74.233 (5)°
β = 86.461 (5)°
γ = 86.845 (5)°
*V* = 1582.0 (10) Å^3^
*Z* = 4
Mo *K*α radiation
μ = 0.11 mm^−1^
*T* = 293 K
0.30 × 0.20 × 0.20 mm

#### Data collection

Bruker SMART APEX CCD area-detector diffractometer
Absorption correction: multi-scan (*SADABS*; Sheldrick, 1996 ▶) *T*_min_ = 0.967, *T*_max_ = 0.978
7698 measured reflections
5590 independent reflections
3283 reflections with *I* > 2σ(*I*)
*R*_int_ = 0.042

#### Refinement

*R*[*F* ^2^ > 2σ(*F* ^2^)] = 0.061
*wR*(*F* ^2^) = 0.171
*S* = 0.96
5590 reflections
481 parameters
6 restraints
H-atom parameters constrained
Δρ_max_ = 0.25 e Å^−3^
Δρ_min_ = −0.35 e Å^−3^

### 

Data collection: *SMART* (Bruker, 2005 ▶); cell refinement: *SAINT* (Bruker, 2005 ▶); data reduction: *SAINT*; program(s) used to solve structure: *SHELXS97* (Sheldrick, 2008 ▶); program(s) used to refine structure: *SHELXL97* (Sheldrick, 2008 ▶); molecular graphics: *SHELXTL* (Sheldrick, 2008 ▶); software used to prepare material for publication: *SHELXL97*.

## Supplementary Material

Crystal structure: contains datablocks I, global. DOI: 10.1107/S1600536809050715/pk2205sup1.cif

Structure factors: contains datablocks I. DOI: 10.1107/S1600536809050715/pk2205Isup2.hkl

Additional supplementary materials:  crystallographic information; 3D view; checkCIF report

## Figures and Tables

**Table 1:** Hydrogen-bond geometry (Å, °)

*D*—H⋯*A*	*D*—H	H⋯*A*	*D*⋯*A*	*D*—H⋯*A*
O3—H3⋯O16^i^	0.82	2.06	2.806 (3)	152
O3—H3⋯O2*A*	0.82	2.36	2.772 (5)	112
O3—H3⋯O2*B*	0.82	2.37	2.799 (9)	114
O5—H5⋯O6	0.82	1.86	2.586 (3)	146
O13—H1⋯O5^ii^	0.82	2.05	2.825 (3)	158
O13—H1⋯O14	0.82	2.29	2.736 (3)	115
O15—H2⋯O16	0.82	1.88	2.600 (3)	147

Symmetry codes: (i) 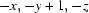; (ii) 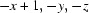.

**Fig. 1.**
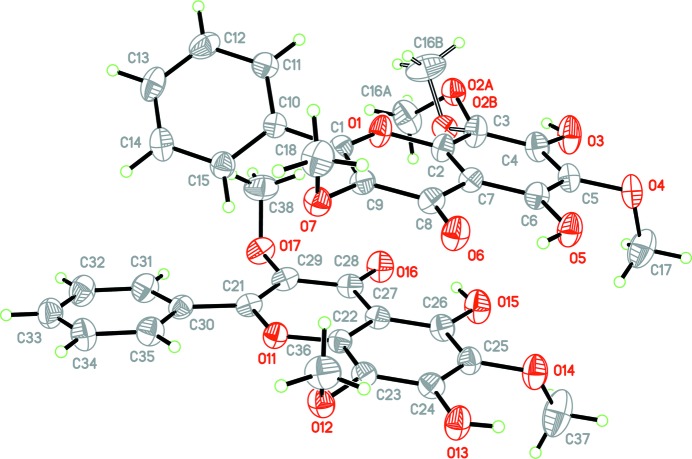
The molecular structure showing the atom-labelling scheme with displacement ellipsoids drawn at the 30% probability level. H atoms are shown as small spheres of arbitrary radius.

## supplementary crystallographic information

### Comment

*Ainsliaea henryi* Diels is mainly distributed in the south-west of China. The whole plant of *Ainsliaea henryi* has been used in Chinese folk medicine to treat cough, asthma and lumbago (Chinese Materia Medica, 2007). The chemical constituents of this plant have not all been reported previously. Our chemical investigation of this plant for bioactive components resulted in the isolation of the title compound, which was previously obtained from the flowers of *Gnaphalium elegans *(Torrenegra *et al.*, 1980). The molecular structure is shown in Fig. 1. Bond lengths and angles are within normal ranges.

### Experimental

The dry powders (5 kg) of the whole plant of *Ainsliaea henryi* were refluxed for 1 h with 95% ethanol (50*L*) three times. After removal of the ethanol under reduced pressure, the extract was suspended in water and then partitioned with petroleum ether, chloroform, ethyl acetate and n-butanol. The chloroform soluble fraction (30 g) was subjected to silica gel column chromatography using gradient elution (petroleum ether/acetone, 15:1 to 2:1, *v*/*v*). 5,7-Dihydroxy-3,6,8-trimethoxyflavone was obtained from the fraction eluted by petroleum ether/acetone (2:1). Single crystals suitable for X-ray diffraction analysis were obtained by slow evaporation from acetone after two weeks at room temperature.

### Refinement

The hydroxyl H atoms attached to O2 was located in a difference Fourier map and refined isotropically with a constraint distance 0.82 Å to the related oxygen atoms. The remaining H atoms were placed in calculated positions with C—H distances in the range 0.93–0.98 Å. The *U*_iso_ values were set equal to 1.5*U*_eq_ (C,O) for methyl and hydroxyl H atoms and 1.2*U*_eq_(C) for the remaining H atoms.

### Crystal data

**Table:**

C_18_H_16_O_7_	*Z* = 4
*M**_r_* = 344.31	*F*(000) = 720
Triclinic, *P*1	*D*_x_ = 1.446 Mg m^−^^3^
Hall symbol: -P 1	Mo *K*α radiation, λ = 0.71073 Å
*a* = 10.147 (4) Å	Cell parameters from 688 reflections
*b* = 11.493 (4) Å	θ = 2.7–25.1°
*c* = 14.134 (5) Å	µ = 0.11 mm^−^^1^
α = 74.233 (5)°	*T* = 293 K
β = 86.461 (5)°	Block, yellow
γ = 86.845 (5)°	0.30 × 0.20 × 0.20 mm
*V* = 1582.0 (10) Å^3^	

### Data collection

**Table:**

Bruker SMART APEX CCD area-detector diffractometer	5590 independent reflections
Radiation source: fine-focus sealed tube	3283 reflections with *I* > 2σ(*I*)
graphite	*R*_int_ = 0.042
φ and ω scans	θ_max_ = 25.2°, θ_min_ = 1.5°
Absorption correction: multi-scan (*SADABS*; Sheldrick, 1996)	*h* = −12→12
*T*_min_ = 0.967, *T*_max_ = 0.978	*k* = −13→13
7698 measured reflections	*l* = −16→9

### Refinement

**Table:**

Refinement on *F*^2^	Primary atom site location: structure-invariant direct methods
Least-squares matrix: full	Secondary atom site location: difference Fourier map
*R*[*F*^2^ > 2σ(*F*^2^)] = 0.061	Hydrogen site location: inferred from neighbouring sites
*wR*(*F*^2^) = 0.171	H-atom parameters constrained
*S* = 0.96	*w* = 1/[σ^2^(*F*_o_^2^) + (0.0916*P*)^2^] where *P* = (*F*_o_^2^ + 2*F*_c_^2^)/3
5590 reflections	(Δ/σ)_max_ < 0.001
481 parameters	Δρ_max_ = 0.25 e Å^−^^3^
6 restraints	Δρ_min_ = −0.35 e Å^−^^3^
0 constraints	

### Special details

**Table:**

Geometry. All e.s.d.'s (except the e.s.d. in the dihedral angle between two l.s. planes) are estimated using the full covariance matrix. The cell e.s.d.'s are taken into account individually in the estimation of e.s.d.'s in distances, angles and torsion angles; correlations between e.s.d.'s in cell parameters are only used when they are defined by crystal symmetry. An approximate (isotropic) treatment of cell e.s.d.s is used for estimating e.s.d.'s involving l.s. planes.
Refinement. Refinement of *F*^2^ against ALL reflections. The weighted *R*-factor *wR* and goodness of fit *S* are based on *F*^2^, conventional *R*-factors *R* are based on *F*, with *F* set to zero for negative *F*^2^. The threshold expression of *F*^2^ > 2σ(*F*^2^) is used only for calculating *R*-factors(gt) *etc*. and is not relevant to the choice of reflections for refinement. *R*-factors based on *F*^2^ are statistically about twice as large as those based on *F*, and *R*- factors based on ALL data will be even larger.

### Fractional atomic coordinates and isotropic or equivalent isotropic displacement parameters (Å^2^)

**Table:**

	*x*	*y*	*z*	*U*_iso_*/*U*_eq_	Occ. (<1)
O2A	0.2156 (5)	0.6030 (5)	0.0119 (3)	0.0541 (13)	0.681 (9)
C16A	0.1025 (6)	0.5890 (7)	0.0772 (5)	0.081 (2)	0.681 (9)
H16A	0.0614	0.5153	0.0787	0.121*	0.681 (9)
H16B	0.0412	0.6563	0.0550	0.121*	0.681 (9)
H16C	0.1282	0.5862	0.1420	0.121*	0.681 (9)
O2B	0.1539 (9)	0.5492 (8)	0.0303 (7)	0.054 (3)	0.319 (9)
C16B	0.1933 (16)	0.6617 (11)	0.0412 (15)	0.091 (5)	0.319 (9)
H16D	0.2672	0.6479	0.0827	0.136*	0.319 (9)
H16E	0.1211	0.6993	0.0706	0.136*	0.319 (9)
H16F	0.2181	0.7137	−0.0222	0.136*	0.319 (9)
O1	0.33194 (18)	0.44978 (17)	0.17638 (13)	0.0552 (5)	
O3	0.2267 (2)	0.5264 (2)	−0.15835 (14)	0.0758 (7)	
H3	0.1715	0.5729	−0.1418	0.114*	
O4	0.39213 (19)	0.34365 (19)	−0.18117 (13)	0.0630 (6)	
O5	0.52258 (19)	0.20267 (18)	−0.02531 (14)	0.0594 (5)	
H5	0.5487	0.1629	0.0281	0.089*	
O6	0.57099 (19)	0.15879 (17)	0.15881 (14)	0.0604 (6)	
O7	0.52849 (17)	0.21483 (16)	0.33769 (13)	0.0507 (5)	
C1	0.3942 (2)	0.3804 (2)	0.25738 (19)	0.0439 (6)	
C2	0.3477 (3)	0.4194 (2)	0.0884 (2)	0.0485 (7)	
C3	0.2788 (3)	0.4914 (3)	0.0111 (2)	0.0583 (8)	
C4	0.2931 (3)	0.4636 (3)	−0.0788 (2)	0.0553 (7)	
C5	0.3765 (3)	0.3673 (3)	−0.09125 (19)	0.0488 (7)	
C6	0.4439 (2)	0.2966 (2)	−0.0127 (2)	0.0463 (7)	
C7	0.4305 (2)	0.3227 (2)	0.07893 (19)	0.0423 (6)	
C8	0.4973 (2)	0.2472 (2)	0.1637 (2)	0.0451 (6)	
C9	0.4720 (2)	0.2829 (2)	0.25352 (19)	0.0430 (6)	
C10	0.3644 (2)	0.4280 (2)	0.34344 (19)	0.0413 (6)	
C11	0.3612 (3)	0.5518 (2)	0.3333 (2)	0.0524 (7)	
H11	0.3771	0.6056	0.2718	0.063*	
C12	0.3346 (3)	0.5944 (3)	0.4150 (3)	0.0644 (9)	
H12	0.3342	0.6770	0.4087	0.077*	
C13	0.3085 (3)	0.5153 (3)	0.5058 (3)	0.0695 (9)	
H13	0.2911	0.5447	0.5607	0.083*	
C14	0.3080 (3)	0.3933 (3)	0.5155 (2)	0.0652 (9)	
H14	0.2881	0.3403	0.5767	0.078*	
C15	0.3369 (3)	0.3492 (3)	0.4351 (2)	0.0492 (7)	
H15	0.3380	0.2663	0.4422	0.059*	
C17	0.2965 (3)	0.2655 (4)	−0.1966 (3)	0.0887 (12)	
H17A	0.2953	0.1933	−0.1430	0.133*	
H17B	0.3188	0.2446	−0.2571	0.133*	
H17C	0.2108	0.3058	−0.2002	0.133*	
C18	0.6633 (3)	0.2394 (3)	0.3434 (2)	0.0677 (9)	
H18A	0.7160	0.2122	0.2942	0.102*	
H18B	0.6930	0.1976	0.4074	0.102*	
H18C	0.6719	0.3248	0.3326	0.102*	
O11	0.17209 (16)	0.07702 (15)	0.40008 (13)	0.0471 (5)	
O12	0.34604 (18)	−0.09955 (16)	0.38200 (13)	0.0538 (5)	
O13	0.4014 (2)	−0.13712 (18)	0.20014 (14)	0.0615 (6)	
H1	0.4080	−0.1414	0.1431	0.092*	
O14	0.28707 (19)	0.00309 (19)	0.03475 (14)	0.0652 (6)	
O15	0.1047 (2)	0.1880 (2)	0.05288 (14)	0.0686 (6)	
H2	0.0590	0.2418	0.0681	0.103*	
O17	−0.06171 (16)	0.33282 (17)	0.35675 (14)	0.0568 (5)	
O16	−0.01670 (19)	0.30149 (18)	0.17101 (14)	0.0652 (6)	
C21	0.0844 (2)	0.1638 (2)	0.4174 (2)	0.0449 (7)	
C22	0.2006 (2)	0.0650 (2)	0.30716 (19)	0.0427 (6)	
C23	0.2919 (2)	−0.0251 (2)	0.29875 (19)	0.0435 (6)	
C24	0.3162 (2)	−0.0457 (2)	0.2067 (2)	0.0478 (7)	
C25	0.2532 (3)	0.0272 (3)	0.1242 (2)	0.0502 (7)	
C26	0.1650 (3)	0.1182 (3)	0.1336 (2)	0.0496 (7)	
C27	0.1367 (2)	0.1396 (2)	0.22674 (19)	0.0435 (6)	
C28	0.0448 (2)	0.2327 (2)	0.2411 (2)	0.0476 (7)	
C29	0.0255 (2)	0.2440 (2)	0.3408 (2)	0.0476 (7)	
C30	0.0705 (2)	0.1546 (2)	0.5235 (2)	0.0470 (7)	
C31	−0.0251 (3)	0.2212 (3)	0.5644 (2)	0.0623 (8)	
H31	−0.0833	0.2744	0.5237	0.075*	
C32	−0.0339 (3)	0.2089 (3)	0.6642 (2)	0.0686 (9)	
H32	−0.0974	0.2546	0.6903	0.082*	
C33	0.0498 (3)	0.1302 (3)	0.7255 (2)	0.0675 (9)	
H33	0.0432	0.1225	0.7928	0.081*	
C34	0.1440 (3)	0.0619 (3)	0.6870 (2)	0.0675 (9)	
H34	0.2010	0.0081	0.7284	0.081*	
C35	0.1534 (3)	0.0740 (3)	0.5870 (2)	0.0590 (8)	
H35	0.2166	0.0273	0.5617	0.071*	
C36	0.4841 (3)	−0.0855 (3)	0.3886 (2)	0.0706 (9)	
H36A	0.5339	−0.1244	0.3451	0.106*	
H36B	0.5090	−0.1215	0.4550	0.106*	
H36C	0.5022	−0.0009	0.3703	0.106*	
C37	0.1887 (4)	−0.0458 (4)	−0.0052 (3)	0.1063 (14)	
H37A	0.1590	−0.1180	0.0416	0.159*	
H37B	0.2233	−0.0650	−0.0641	0.159*	
H37C	0.1158	0.0119	−0.0206	0.159*	
C38	−0.0057 (3)	0.4495 (3)	0.3365 (3)	0.0771 (10)	
H38A	0.0523	0.4502	0.3875	0.116*	
H38B	−0.0752	0.5102	0.3341	0.116*	
H38C	0.0433	0.4662	0.2743	0.116*	

### Atomic displacement parameters (Å^2^)

**Table:**

	*U*^11^	*U*^22^	*U*^33^	*U*^12^	*U*^13^	*U*^23^
O2A	0.065 (3)	0.046 (3)	0.046 (2)	0.014 (2)	−0.0035 (18)	−0.0042 (19)
C16A	0.073 (4)	0.081 (5)	0.073 (4)	0.025 (4)	0.012 (3)	−0.004 (3)
O2B	0.049 (6)	0.053 (5)	0.056 (5)	0.001 (4)	−0.002 (4)	−0.011 (4)
C16B	0.080 (11)	0.056 (9)	0.144 (14)	0.010 (8)	−0.032 (10)	−0.037 (10)
O1	0.0649 (12)	0.0613 (12)	0.0418 (11)	0.0228 (10)	−0.0135 (9)	−0.0206 (9)
O3	0.0831 (16)	0.0953 (17)	0.0435 (12)	0.0396 (13)	−0.0134 (11)	−0.0153 (11)
O4	0.0627 (13)	0.0872 (16)	0.0391 (12)	0.0051 (11)	0.0069 (9)	−0.0210 (11)
O5	0.0621 (12)	0.0675 (14)	0.0502 (12)	0.0222 (10)	−0.0046 (10)	−0.0231 (11)
O6	0.0674 (13)	0.0582 (13)	0.0559 (12)	0.0258 (11)	−0.0103 (10)	−0.0198 (10)
O7	0.0533 (11)	0.0500 (11)	0.0460 (11)	0.0072 (9)	−0.0111 (9)	−0.0081 (9)
C1	0.0457 (15)	0.0435 (16)	0.0430 (16)	0.0035 (13)	−0.0115 (12)	−0.0116 (13)
C2	0.0491 (16)	0.0544 (17)	0.0439 (17)	0.0087 (13)	−0.0065 (13)	−0.0178 (14)
C3	0.0642 (19)	0.064 (2)	0.0463 (18)	0.0266 (16)	−0.0108 (14)	−0.0185 (15)
C4	0.0522 (17)	0.064 (2)	0.0446 (17)	0.0107 (15)	−0.0066 (14)	−0.0086 (15)
C5	0.0432 (15)	0.0645 (19)	0.0382 (16)	0.0014 (14)	−0.0005 (12)	−0.0140 (14)
C6	0.0389 (14)	0.0518 (17)	0.0488 (17)	0.0053 (13)	−0.0018 (12)	−0.0160 (14)
C7	0.0401 (14)	0.0438 (15)	0.0434 (16)	0.0012 (12)	−0.0061 (12)	−0.0122 (12)
C8	0.0414 (15)	0.0434 (16)	0.0510 (17)	0.0056 (13)	−0.0070 (12)	−0.0138 (13)
C9	0.0442 (15)	0.0423 (15)	0.0421 (16)	0.0009 (12)	−0.0117 (12)	−0.0092 (12)
C10	0.0368 (14)	0.0470 (16)	0.0407 (15)	0.0022 (12)	−0.0034 (11)	−0.0135 (13)
C11	0.0503 (16)	0.0504 (17)	0.0556 (18)	−0.0009 (13)	0.0001 (13)	−0.0138 (14)
C12	0.0611 (19)	0.058 (2)	0.085 (3)	−0.0021 (15)	0.0023 (17)	−0.039 (2)
C13	0.072 (2)	0.088 (3)	0.061 (2)	0.0083 (18)	0.0002 (17)	−0.045 (2)
C14	0.072 (2)	0.079 (2)	0.0424 (18)	0.0060 (17)	0.0007 (15)	−0.0148 (16)
C15	0.0513 (16)	0.0505 (17)	0.0456 (17)	0.0030 (13)	−0.0058 (13)	−0.0128 (14)
C17	0.084 (2)	0.124 (3)	0.077 (3)	0.006 (2)	−0.018 (2)	−0.058 (2)
C18	0.0559 (19)	0.082 (2)	0.066 (2)	0.0076 (17)	−0.0229 (16)	−0.0194 (17)
O11	0.0491 (10)	0.0463 (11)	0.0465 (11)	0.0086 (9)	−0.0069 (8)	−0.0144 (9)
O12	0.0581 (12)	0.0522 (11)	0.0445 (11)	0.0141 (9)	−0.0080 (9)	−0.0037 (9)
O13	0.0733 (13)	0.0622 (13)	0.0485 (12)	0.0293 (11)	−0.0090 (10)	−0.0188 (10)
O14	0.0605 (13)	0.0839 (15)	0.0515 (13)	0.0176 (11)	−0.0041 (10)	−0.0227 (11)
O15	0.0735 (15)	0.0762 (15)	0.0498 (12)	0.0290 (11)	−0.0159 (11)	−0.0095 (11)
O17	0.0416 (10)	0.0568 (13)	0.0751 (14)	0.0130 (9)	−0.0060 (9)	−0.0251 (11)
O16	0.0634 (13)	0.0673 (14)	0.0600 (13)	0.0266 (11)	−0.0135 (10)	−0.0120 (11)
C21	0.0351 (14)	0.0437 (16)	0.0577 (18)	0.0010 (12)	−0.0053 (13)	−0.0165 (14)
C22	0.0413 (14)	0.0433 (15)	0.0439 (16)	−0.0026 (12)	−0.0056 (12)	−0.0112 (13)
C23	0.0447 (15)	0.0409 (15)	0.0426 (16)	0.0045 (12)	−0.0101 (12)	−0.0066 (12)
C24	0.0456 (16)	0.0443 (16)	0.0504 (18)	0.0052 (13)	−0.0058 (13)	−0.0079 (13)
C25	0.0519 (17)	0.0545 (18)	0.0427 (17)	0.0053 (14)	−0.0034 (13)	−0.0121 (14)
C26	0.0464 (16)	0.0540 (17)	0.0452 (17)	0.0060 (14)	−0.0107 (13)	−0.0075 (13)
C27	0.0398 (14)	0.0418 (15)	0.0468 (16)	0.0014 (12)	−0.0069 (12)	−0.0078 (12)
C28	0.0404 (15)	0.0457 (16)	0.0547 (18)	0.0037 (13)	−0.0122 (13)	−0.0088 (14)
C29	0.0354 (14)	0.0453 (16)	0.0653 (19)	0.0042 (12)	−0.0078 (13)	−0.0200 (14)
C30	0.0413 (15)	0.0505 (17)	0.0528 (18)	−0.0055 (13)	−0.0010 (13)	−0.0194 (14)
C31	0.0537 (18)	0.070 (2)	0.067 (2)	0.0045 (15)	−0.0012 (15)	−0.0253 (17)
C32	0.061 (2)	0.086 (2)	0.065 (2)	−0.0013 (18)	0.0100 (17)	−0.0343 (19)
C33	0.070 (2)	0.084 (2)	0.053 (2)	−0.0173 (19)	0.0077 (17)	−0.0248 (18)
C34	0.075 (2)	0.077 (2)	0.048 (2)	0.0015 (18)	−0.0066 (16)	−0.0123 (17)
C35	0.0560 (18)	0.061 (2)	0.061 (2)	0.0049 (15)	−0.0029 (15)	−0.0196 (16)
C36	0.067 (2)	0.067 (2)	0.074 (2)	0.0077 (17)	−0.0307 (17)	−0.0090 (17)
C37	0.077 (3)	0.174 (4)	0.091 (3)	0.020 (3)	−0.019 (2)	−0.077 (3)
C38	0.070 (2)	0.050 (2)	0.111 (3)	0.0058 (17)	0.0011 (19)	−0.0235 (19)

### Geometric parameters (Å, °)

**Table:**

O2A—C3	1.404 (5)	C18—H18B	0.9599
O2A—C16A	1.416 (7)	C18—H18C	0.9599
C16A—H16A	0.9599	O11—C21	1.362 (3)
C16A—H16B	0.9599	O11—C22	1.369 (3)
C16A—H16C	0.9599	O12—C23	1.378 (3)
O2B—C16B	1.425 (13)	O12—C36	1.431 (3)
O2B—C3	1.444 (8)	O13—C24	1.343 (3)
C16B—H16D	0.9599	O13—H1	0.8200
C16B—H16E	0.9599	O14—C25	1.386 (3)
C16B—H16F	0.9599	O14—C37	1.391 (4)
O1—C1	1.372 (3)	O15—C26	1.361 (3)
O1—C2	1.378 (3)	O15—H2	0.8200
O3—C4	1.356 (3)	O17—C29	1.370 (3)
O3—H3	0.8200	O17—C38	1.435 (3)
O4—C5	1.368 (3)	O16—C28	1.262 (3)
O4—C17	1.423 (4)	C21—C29	1.363 (4)
O5—C6	1.352 (3)	C21—C30	1.473 (4)
O5—H5	0.8200	C22—C23	1.376 (3)
O6—C8	1.243 (3)	C22—C27	1.395 (4)
O7—C9	1.373 (3)	C23—C24	1.389 (3)
O7—C18	1.423 (3)	C24—C25	1.404 (4)
C1—C9	1.346 (3)	C25—C26	1.367 (4)
C1—C10	1.473 (3)	C26—C27	1.414 (4)
C2—C3	1.381 (4)	C27—C28	1.429 (4)
C2—C7	1.387 (3)	C28—C29	1.450 (4)
C3—C4	1.390 (4)	C30—C35	1.388 (4)
C4—C5	1.398 (4)	C30—C31	1.396 (4)
C5—C6	1.379 (4)	C31—C32	1.378 (4)
C6—C7	1.404 (3)	C31—H31	0.9300
C7—C8	1.456 (4)	C32—C33	1.367 (5)
C8—C9	1.441 (3)	C32—H32	0.9300
C10—C15	1.386 (4)	C33—C34	1.382 (4)
C10—C11	1.390 (4)	C33—H33	0.9300
C11—C12	1.378 (4)	C34—C35	1.380 (4)
C11—H11	0.9300	C34—H34	0.9300
C12—C13	1.376 (4)	C35—H35	0.9300
C12—H12	0.9300	C36—H36A	0.9599
C13—C14	1.372 (4)	C36—H36B	0.9599
C13—H13	0.9300	C36—H36C	0.9599
C14—C15	1.375 (4)	C37—H37A	0.9599
C14—H14	0.9300	C37—H37B	0.9599
C15—H15	0.9300	C37—H37C	0.9599
C17—H17A	0.9599	C38—H38A	0.9599
C17—H17B	0.9599	C38—H38B	0.9599
C17—H17C	0.9599	C38—H38C	0.9599
C18—H18A	0.9599		
			
C3—O2A—C16A	112.2 (6)	H18B—C18—H18C	109.5
C16B—O2B—C3	102.2 (11)	C21—O11—C22	121.8 (2)
O2B—C16B—H16D	109.5	C23—O12—C36	114.4 (2)
O2B—C16B—H16E	109.5	C24—O13—H1	109.5
H16D—C16B—H16E	109.5	C25—O14—C37	115.6 (2)
O2B—C16B—H16F	109.5	C26—O15—H2	109.5
H16D—C16B—H16F	109.5	C29—O17—C38	113.8 (2)
H16E—C16B—H16F	109.5	O11—C21—C29	119.9 (2)
C1—O1—C2	119.78 (19)	O11—C21—C30	110.4 (2)
C4—O3—H3	109.5	C29—C21—C30	129.7 (2)
C5—O4—C17	113.5 (2)	O11—C22—C23	116.5 (2)
C6—O5—H5	109.5	O11—C22—C27	120.7 (2)
C9—O7—C18	113.8 (2)	C23—C22—C27	122.8 (2)
C9—C1—O1	121.8 (2)	C22—C23—O12	119.7 (2)
C9—C1—C10	127.0 (2)	C22—C23—C24	118.1 (2)
O1—C1—C10	111.2 (2)	O12—C23—C24	121.9 (2)
O1—C2—C3	116.3 (2)	O13—C24—C23	117.6 (2)
O1—C2—C7	121.0 (2)	O13—C24—C25	121.8 (2)
C3—C2—C7	122.6 (2)	C23—C24—C25	120.6 (2)
C2—C3—C4	117.8 (2)	C26—C25—O14	122.8 (2)
C2—C3—O2A	124.0 (3)	C26—C25—C24	120.5 (2)
C4—C3—O2A	117.1 (3)	O14—C25—C24	116.7 (2)
C2—C3—O2B	119.9 (4)	O15—C26—C25	119.7 (2)
C4—C3—O2B	115.5 (4)	O15—C26—C27	120.2 (2)
O3—C4—C3	122.4 (2)	C25—C26—C27	120.0 (2)
O3—C4—C5	116.5 (2)	C22—C27—C26	117.9 (2)
C3—C4—C5	121.2 (3)	C22—C27—C28	119.6 (2)
O4—C5—C6	120.0 (2)	C26—C27—C28	122.5 (2)
O4—C5—C4	120.2 (2)	O16—C28—C27	122.0 (3)
C6—C5—C4	119.8 (2)	O16—C28—C29	121.6 (2)
O5—C6—C5	119.3 (2)	C27—C28—C29	116.4 (2)
O5—C6—C7	120.7 (2)	C21—C29—O17	120.7 (3)
C5—C6—C7	120.0 (2)	C21—C29—C28	121.4 (2)
C2—C7—C6	118.6 (2)	O17—C29—C28	117.8 (2)
C2—C7—C8	120.3 (2)	C35—C30—C31	117.8 (3)
C6—C7—C8	121.1 (2)	C35—C30—C21	119.1 (2)
O6—C8—C9	122.6 (2)	C31—C30—C21	123.1 (3)
O6—C8—C7	122.4 (2)	C32—C31—C30	120.7 (3)
C9—C8—C7	115.0 (2)	C32—C31—H31	119.7
C1—C9—O7	119.2 (2)	C30—C31—H31	119.7
C1—C9—C8	122.0 (2)	C33—C32—C31	120.7 (3)
O7—C9—C8	118.8 (2)	C33—C32—H32	119.7
C15—C10—C11	119.5 (2)	C31—C32—H32	119.7
C15—C10—C1	120.0 (2)	C32—C33—C34	119.7 (3)
C11—C10—C1	120.5 (2)	C32—C33—H33	120.1
C12—C11—C10	119.5 (3)	C34—C33—H33	120.1
C12—C11—H11	120.2	C35—C34—C33	119.8 (3)
C10—C11—H11	120.2	C35—C34—H34	120.1
C13—C12—C11	120.5 (3)	C33—C34—H34	120.1
C13—C12—H12	119.8	C34—C35—C30	121.2 (3)
C11—C12—H12	119.8	C34—C35—H35	119.4
C14—C13—C12	120.1 (3)	C30—C35—H35	119.4
C14—C13—H13	120.0	O12—C36—H36A	109.5
C12—C13—H13	120.0	O12—C36—H36B	109.5
C13—C14—C15	120.2 (3)	H36A—C36—H36B	109.5
C13—C14—H14	119.9	O12—C36—H36C	109.5
C15—C14—H14	119.9	H36A—C36—H36C	109.5
C14—C15—C10	120.2 (3)	H36B—C36—H36C	109.5
C14—C15—H15	119.9	O14—C37—H37A	109.5
C10—C15—H15	119.9	O14—C37—H37B	109.5
O4—C17—H17A	109.5	H37A—C37—H37B	109.5
O4—C17—H17B	109.5	O14—C37—H37C	109.5
H17A—C17—H17B	109.5	H37A—C37—H37C	109.5
O4—C17—H17C	109.5	H37B—C37—H37C	109.5
H17A—C17—H17C	109.5	O17—C38—H38A	109.5
H17B—C17—H17C	109.5	O17—C38—H38B	109.5
O7—C18—H18A	109.5	H38A—C38—H38B	109.5
O7—C18—H18B	109.5	O17—C38—H38C	109.5
H18A—C18—H18B	109.5	H38A—C38—H38C	109.5
O7—C18—H18C	109.5	H38B—C38—H38C	109.5
H18A—C18—H18C	109.5		
			
C2—O1—C1—C9	0.5 (4)	C12—C13—C14—C15	1.6 (5)
C2—O1—C1—C10	−179.9 (2)	C13—C14—C15—C10	−1.1 (4)
C1—O1—C2—C3	178.1 (3)	C11—C10—C15—C14	−0.6 (4)
C1—O1—C2—C7	−2.9 (4)	C1—C10—C15—C14	−179.5 (2)
O1—C2—C3—C4	179.7 (3)	C22—O11—C21—C29	−2.0 (4)
C7—C2—C3—C4	0.8 (5)	C22—O11—C21—C30	178.8 (2)
O1—C2—C3—O2A	12.1 (5)	C21—O11—C22—C23	179.2 (2)
C7—C2—C3—O2A	−166.8 (4)	C21—O11—C22—C27	−2.2 (3)
O1—C2—C3—O2B	−30.4 (6)	O11—C22—C23—O12	2.0 (4)
C7—C2—C3—O2B	150.6 (5)	C27—C22—C23—O12	−176.6 (2)
C16A—O2A—C3—C2	−70.4 (7)	O11—C22—C23—C24	175.4 (2)
C16A—O2A—C3—C4	121.9 (5)	C27—C22—C23—C24	−3.1 (4)
C16A—O2A—C3—O2B	25.1 (6)	C36—O12—C23—C22	−113.0 (3)
C16B—O2B—C3—C2	90.0 (11)	C36—O12—C23—C24	73.8 (3)
C16B—O2B—C3—C4	−119.5 (9)	C22—C23—C24—O13	−177.3 (2)
C16B—O2B—C3—O2A	−17.8 (8)	O12—C23—C24—O13	−4.0 (4)
C2—C3—C4—O3	178.0 (3)	C22—C23—C24—C25	2.7 (4)
O2A—C3—C4—O3	−13.5 (5)	O12—C23—C24—C25	176.0 (2)
O2B—C3—C4—O3	26.8 (6)	C37—O14—C25—C26	−70.2 (4)
C2—C3—C4—C5	−1.3 (5)	C37—O14—C25—C24	111.0 (3)
O2A—C3—C4—C5	167.2 (3)	O13—C24—C25—C26	178.8 (3)
O2B—C3—C4—C5	−152.5 (5)	C23—C24—C25—C26	−1.2 (4)
C17—O4—C5—C6	92.7 (3)	O13—C24—C25—O14	−2.4 (4)
C17—O4—C5—C4	−87.7 (3)	C23—C24—C25—O14	177.6 (2)
O3—C4—C5—O4	2.6 (4)	O14—C25—C26—O15	1.3 (4)
C3—C4—C5—O4	−178.1 (3)	C24—C25—C26—O15	−180.0 (3)
O3—C4—C5—C6	−177.8 (3)	O14—C25—C26—C27	−178.7 (2)
C3—C4—C5—C6	1.6 (4)	C24—C25—C26—C27	0.0 (4)
O4—C5—C6—O5	−1.4 (4)	O11—C22—C27—C26	−176.5 (2)
C4—C5—C6—O5	178.9 (2)	C23—C22—C27—C26	2.0 (4)
O4—C5—C6—C7	178.4 (2)	O11—C22—C27—C28	2.6 (4)
C4—C5—C6—C7	−1.2 (4)	C23—C22—C27—C28	−178.9 (2)
O1—C2—C7—C6	−179.4 (2)	O15—C26—C27—C22	179.6 (2)
C3—C2—C7—C6	−0.5 (4)	C25—C26—C27—C22	−0.3 (4)
O1—C2—C7—C8	3.2 (4)	O15—C26—C27—C28	0.5 (4)
C3—C2—C7—C8	−177.9 (3)	C25—C26—C27—C28	−179.5 (2)
O5—C6—C7—C2	−179.5 (2)	C22—C27—C28—O16	−179.1 (2)
C5—C6—C7—C2	0.7 (4)	C26—C27—C28—O16	0.0 (4)
O5—C6—C7—C8	−2.1 (4)	C22—C27—C28—C29	0.8 (4)
C5—C6—C7—C8	178.1 (2)	C26—C27—C28—C29	179.9 (2)
C2—C7—C8—O6	179.1 (2)	O11—C21—C29—O17	−179.2 (2)
C6—C7—C8—O6	1.7 (4)	C30—C21—C29—O17	−0.1 (4)
C2—C7—C8—C9	−1.1 (4)	O11—C21—C29—C28	5.6 (4)
C6—C7—C8—C9	−178.5 (2)	C30—C21—C29—C28	−175.3 (2)
O1—C1—C9—O7	−178.1 (2)	C38—O17—C29—C21	101.0 (3)
C10—C1—C9—O7	2.4 (4)	C38—O17—C29—C28	−83.6 (3)
O1—C1—C9—C8	1.6 (4)	O16—C28—C29—C21	175.0 (3)
C10—C1—C9—C8	−177.9 (2)	C27—C28—C29—C21	−4.9 (4)
C18—O7—C9—C1	−100.6 (3)	O16—C28—C29—O17	−0.3 (4)
C18—O7—C9—C8	79.7 (3)	C27—C28—C29—O17	179.8 (2)
O6—C8—C9—C1	178.5 (3)	O11—C21—C30—C35	7.0 (3)
C7—C8—C9—C1	−1.2 (4)	C29—C21—C30—C35	−172.1 (3)
O6—C8—C9—O7	−1.8 (4)	O11—C21—C30—C31	−171.4 (2)
C7—C8—C9—O7	178.4 (2)	C29—C21—C30—C31	9.5 (4)
C9—C1—C10—C15	−42.8 (4)	C35—C30—C31—C32	1.5 (4)
O1—C1—C10—C15	137.6 (2)	C21—C30—C31—C32	179.9 (3)
C9—C1—C10—C11	138.3 (3)	C30—C31—C32—C33	−0.8 (5)
O1—C1—C10—C11	−41.3 (3)	C31—C32—C33—C34	−0.1 (5)
C15—C10—C11—C12	1.8 (4)	C32—C33—C34—C35	0.2 (5)
C1—C10—C11—C12	−179.3 (2)	C33—C34—C35—C30	0.6 (5)
C10—C11—C12—C13	−1.3 (4)	C31—C30—C35—C34	−1.4 (4)
C11—C12—C13—C14	−0.4 (5)	C21—C30—C35—C34	−179.9 (3)

### Hydrogen-bond geometry (Å, °)

**Table:**

*D*—H···*A*	*D*—H	H···*A*	*D*···*A*	*D*—H···*A*
O3—H3···O16^i^	0.82	2.06	2.806 (3)	152
O3—H3···O2A	0.82	2.36	2.772 (5)	112
O3—H3···O2B	0.82	2.37	2.799 (9)	114
O5—H5···O6	0.82	1.86	2.586 (3)	146
O13—H1···O5^ii^	0.82	2.05	2.825 (3)	158
O13—H1···O14	0.82	2.29	2.736 (3)	115
O15—H2···O16	0.82	1.88	2.600 (3)	147

Symmetry codes: (i) −*x*, −*y*+1, −*z*; (ii) −*x*+1, −*y*, −*z*.
